# To bill or not to bill – a cross-sectional study comparing funded and unfunded advance care planning services in German nursing homes

**DOI:** 10.1186/s12913-025-13848-6

**Published:** 2025-12-25

**Authors:** Tanja Schleef, Christopher Berloge, Anna Völkel, Hannes Jacobs, Birte Burger, Jona T Stahmeyer, Anna Levke Brütt, Falk Hoffmann, Stephanie Stiel

**Affiliations:** 1 Institute for General Practice and Palliative Care, Hannover Medical School, Carl-Neuberg-Straße 1, 30625 Hannover, Germany; 2 Junior Research Group for Rehabilitation Sciences, Department of Health Services Research, Carl von Ossietzky Universität Oldenburg, Oldenburg, Germany; 3 Division of Outpatient Care and Pharmacoepidemiology, Department of Health Services Research, Carl von Ossietzky Universität Oldenburg, Oldenburg, Germany; 4 Health Services Research Unit, AOK Niedersachsen, Hannover, Germany; 5 Department of Medical Psychology, University Medical Center Hamburg-Eppendorf, Hamburg, Germany

**Keywords:** Long-term care, Advance care planning, End of life care, Advance directives, Survey

## Abstract

**Background:**

In 2018, § 132g of the German Social Code, Book V (SGB V), came into force, allowing long-term care facilities in Germany to bill statutory health insurance for advance care planning (ACP). The present study examined differences in end-of-life care structures between nursing homes that had obtained approval for billing ACP and those that had not (and do not intend to obtain this approval), as well as the extent to which ACP is available to residents beyond the scope of these billing options.

**Methods:**

A nationwide cross-sectional survey was conducted in 2023 among a random sample of 1,369 German nursing homes. The questionnaire, primarily targeting nursing home management staff, aimed to evaluate hospice and palliative care structures and ACP implementation. This manuscript compares nursing homes that had obtained approval for ACP billing with those that had not received approval and had no intention of applying for it; therefore, a subset of the returned questionnaires was used for analysis.

**Results:**

A total of 330 questionnaires were received (response rate 24%; mostly completed by nursing home management staff), enabling the comparison of 100 nursing homes with ACP billing approval and 68 without. ACP was more prevalent in nursing homes with billing approval (96.0% vs. 36.4%; *p* < 0.001). While nursing homes with approval reported higher proportions of residents with written health care proxies (66.7% vs. 58.7%; *p* = 0.049) and advance directives (68.5% vs. 55.6%; *p* < 0.001), no significant difference was found in the presence of emergency plans (36.0% vs. 37.6%; *p* = 0.782). Furthermore, a substantial rate of at least one of these three precautionary documents was not considered useful in cases of hospitalization or cardiac arrest. Both nursing home groups reported strong collaboration with external palliative care providers, particularly general practitioners.

**Conclusions:**

ACP appears accessible in nursing homes that have obtained approval for billing ACP, as well as in those that have not. Nursing homes with approval have a higher proportion of residents with advance directives. However, the limited presence of emergency plans and concerns about the utility of the three precautionary documents highlight the need for quality assurance in ACP consultation and documentation.

**Supplementary Information:**

The online version contains supplementary material available at 10.1186/s12913-025-13848-6.

## Background

As a consequence of the aging population, the demand for long-term care is rising [1, 2]. In 2022, the proportion of individuals aged 80 years and over residing in nursing homes (NHs) across Europe ranged from 2.3% to 14.6% [3]. Moreover, the median duration between NH admission and death appears to be decreasing. A retrospective nationwide study based in Finland, for instance, found that the average number of individuals receiving NH care in the last 2 years of life decreased from 96.6 days in 1998 to 63.5 days in 2013 [1]. As a result, NHs are increasingly becoming an important setting for end-of-life care and death. This trend has been observed not only in Germany, but also in other countries within and outside Europe [4–7].

At the end of life, individuals are particularly vulnerable, making them susceptible to both underuse and overuse of health care services [8]. In NHs, palliative care for residents at the end of life remains inadequate, especially with regard to physical and emotional distress [9]. Conversely, overuse of emergency services, frequent hospital transfers, and inpatient hospital stays at the end of life pose significant concerns [10]. Hospital admissions are frequent [11, 12] and impose considerable financial strain on the healthcare system, while also negatively impacting residents by increasing the risk of delirium, hospital-acquired infections, and invasive interventions [10, 13]. Moreover, most residents express a preference to die in the NH [14], rather than in a hospital, suggesting that many hospital transfers conflict with their wishes and care preferences.

Advance care planning (ACP) offers a means of empowering residents by enabling them to articulate their individual care preferences. ACP is a structured, ongoing process of communication between trained facilitators, residents, their next of kin, and relevant healthcare providers. Its objective is to facilitate discussion, documentation, and, if necessary, the adjustment of treatment and care preferences for the future [15, 16]. ACP ensures that individuals who lose the capacity to articulate their wishes or make decisions regarding their healthcare continue to receive care aligned with their preferences [15]. To achieve this, broad implementation of ACP at institutional and regional levels is needed.

In Germany, structured ACP consultations in long-term care facilities became eligible for funding under § 132g Book V of the German Social Code (SGB V) – “Health Care Planning for the Last Phase of Life” – in 2018. NHs seeking to obtain approval to bill statutory health insurance for ACP must meet specific requirements as stated in the implementation agreement, including:

- The presence of a qualified ACP facilitator in attendance;
- The integration of ACP into the overall care structure;
- A palliative care strategy, that includes internal networking as well as external networking with healthcare providers in end-of-life care [17].

Following approval, ACP consultations are reimbursed by health insurance funds at a rate equivalent to 0.25 of a facilitator’s full-time position per 100 residents. Currently, ACP is reimbursed on a flat-charge basis, with the number of residents in the respective NH being the determining factor, regardless of whether ACP was actually used [17, 18].

However, research on the implementation of ACP under § 132g SGB V remains limited. By the end of 2022, approximately 15% of NHs had implemented ACP in accordance with this regulation [19]. The reasons for this low uptake, as well as the characteristics distinguishing NHs that obtained approval from NHs that did intentionally not apply for, remain unclear. Therefore, the present study addresses the following two research questions:

1. What differences exist in end-of-life care structures and ACP between NHs that obtained approval under § 132g SGB V and those neither approved nor planning to obtain approval?
2. To what extent are ACP consultations and collaboration with healthcare providers in end-of-life care accessible to residents outside the scope of billing options?

## Methods

### Study design

The present cross-sectional study was based on a questionnaire survey conducted as part of the “Gut-Leben” project. As a mixed-methods study, “Gut-Leben” aims to evaluate the implementation of and barriers to ACP under § 132g SGB V in German NHs and to provide practical recommendations for improving end-of-life care. Study reporting followed the Reporting of Observational Studies in Epidemiology (STROBE) checklist for cross-sectional studies [20]. The study design has been previously reported in detail [21].

The survey utilized the AOK Care Navigator, a database maintained by the Federal Association of Local Health Insurance Funds, which provides a comprehensive and quality-assured dataset on long-term care facilities across Germany. The target population included 11,625 long-term care facilities, encompassing NHs for residents with dementia or psychiatric disorders, as well as those requiring ventilation support. From this pool, a simple random sample of 1,400 NHs was drawn using IBM SPSS Statistics (Version 28) and manually screened for the exclusion criteria. Facilities offering only day care or short-term care were excluded, as ACP consultations in these settings are not reimbursed by health insurance. Furthermore, NH management personnel were identified to enable personalized communication. After verification, 1,369 addresses were included in the final mailing list.

In February 2023, the 1,369 selected NHs were invited by mail to participate in the survey and complete the questionnaire. The questionnaires were sent by post with personalized cover letters and a stamped envelope for the return, which did not require any address details or designation of the participating NH. A follow-up reminder, including another copy of the questionnaire, was sent after 3 weeks. Participation was voluntary, and data collection was completely anonymous.

### Questionnaire

The four-page questionnaire was developed by an interdisciplinary research team and has previously been published as supplementary material [22]. The questionnaire is structured into four sections: (1) end-of-life care, covering healthcare providers involved in NH-based end-of-life care (general practitioners, palliative care physicians, specialized palliative care teams, hospices and voluntary hospice services), the extent of collaboration with these providers, and the availability of ACP; (2) awareness and authorization status regarding the option to bill ACP through statutory health insurance; (3) resident characteristics and medical care, including demographic and healthcare-related information; and (4) NH and respondent characteristics, capturing facility-specific and individual respondent details.

The questionnaire underwent a two-step pilot testing process. First, the project’s practice advisory board reviewed the questionnaire, leading to the refinement of answer categories and terminology. Second, a pretest was conducted with four NH managers using the “think aloud” technique [23], in which participants verbalized their thoughts while answering the questions. This approach helped to identify ambiguities and areas for improvement, resulting in further adjustments to the questions and predefined answer categories.

In the first section, respondents were asked to indicate the availability of key end-of-life care structures in their local region and to rate the intensity of collaboration with healthcare providers on a 5-point Likert scale ranging from 0 (*none*) to 4 (*very strong*). Additionally, they were asked whether their facility offered ACP and, if so, to specify the proportion of residents who had participated in at least one ACP consultation.

The second section began with a single-choice question assessing respondents’ awareness of the legal provision for billing ACP under § 132g SGB V. Those aware of this regulation were further asked whether their NH had obtained approval for ACP billing. If not, respondents were asked whether they had already applied, planned to apply, or had no intention of seeking approval in the future.

To characterize the resident population, respondents were asked to estimate the proportion of residents with an oncological or dementia-related illness, those with a care grade 4 or 5 (the highest levels of care in Germany, representing extremely severe limitations in independence or skills), those who were entirely bedridden, and those who had experienced at least one inpatient hospital stay in the past year. Furthermore, they were asked to indicate the proportion of residents for whom a written health care proxy, an advance directive, or an emergency plan was available. Respondents also assessed the extent to which these documents would be meaningful in the event of hospital transfer during the last phase of life or in the event of cardiac arrest.

Moreover, the questionnaire collected key NH characteristics, including: sponsorship type, location—categorized as rural (≤ 5,000 inhabitants), small town (5,000–20,000 inhabitants), semi-urban (20,000–100,000 inhabitants), or urban (> 100,000 inhabitants)—number of beds, presence of a psychogeriatric ward, and distance to the nearest hospital with an emergency department. In addition, the extent of NH staff training in end-of-life care was surveyed using checkboxes for various types of further education and training programs.

Finally, the respondents were asked to state their age, sex, current position in the NH (nursing home manager, nursing staff manager, managing director or other with the possibility to give a free text answer) and the number of years in their current position.

### Statistical analysis

The study explored the differences between NHs that had obtained approval under § 132g SGB V, and those that had not obtained approval and had no intention of doing so. To enable a valid comparison, only NHs aware of the option to bill ACP consultations under statutory health insurance were included. NHs that were unaware of this option, those for which approval status was unavailable, and those that had already planned or initiated the approval process were excluded from the analysis.

Respondents were categorized into three age groups (≤ 40, 41–60, > 60 years). A qualification in end-of-life care was considered present if at least one individual from the NH management team, staff, or nursing personnel had completed palliative care training (160 h), a basic course in palliative care (40 h), or another continuing education program. The proportion of NHs in which nursing staff had undergone palliative care training was reported separately.

Categorical data were calculated as frequencies (*n*) and proportions (percentages), while continuous data were reported as means with standard deviations (*SD*s), medians, and interquartile ranges (IQRs). Due to missing values, denominators varied across variables.

Group differences by approval status were analyzed using *t*-tests for normally distributed metric variables, such as the number of NH beds. Variables that were not normally distributed, such as resident characteristics, the proportion of ACP consultations, the availability of relevant documents, and the meaningfulness of these documents, as well as the assessment of cooperation in end-of-life care, were compared between approved and not-approved NHs using a Mann-Whitney-U test. Pearson’s χ2-test was employed to compare categorical variables, including respondents’ positions in the NH, the type of sponsorship, qualifications in end-of-life care, and the availability of ACP.

The statistical analyses were conducted using IBM SPSS Statistics version 28 (IBM Corporation, Armonk, NY, USA).

### Ethics

The study was conducted in accordance with the relevant guidelines and regulations from the Declaration of Helsinki and received an ethics waiver from the Medical Ethics Committee of the Carl von Ossietzky Universität Oldenburg (no. 2022–154). As this study evaluated anonymous data that cannot be assigned to a specific person there was no statutory or professional obligation to consult an ethics committee. The participants were informed about the study, the voluntary nature of participation, and the completely anonymous nature of the data collection. The return of the questionnaire via post was considered informed consent.

## Results

**Fig. 1:**
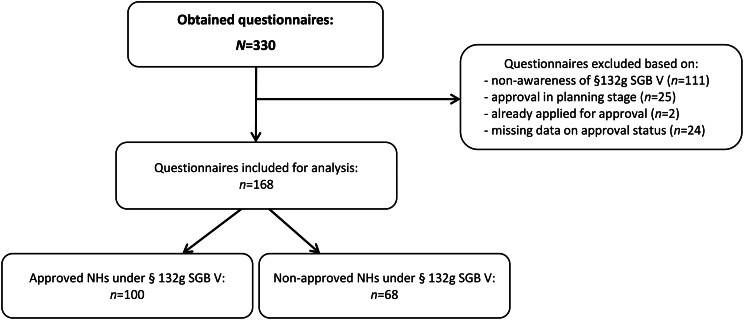
Flowchart of questionnaire inclusion for analysis. SGB V=Book V of the German Social Code, NH=Nursing home

A total of 330 questionnaires were returned, yielding an overall response rate of 24.1%. Of the responding facilities, 111 (33.6%) indicated that they were unaware of the option to bill for ACP through the statutory health insurance scheme and were therefore excluded from further analysis. In addition, 51 NHs were excluded because they either did not provide information on their approval status for ACP billing under § 132g SGB V or they reported that approval was already planned or in progress (see Fig. 1). The final analysis included 100 NHs that had obtained approval under § 132g SGB V, and 68 NHs that were aware of the reimbursement option, but had not obtained this approval and had no intention of applying for it in the future. An analysis of the questionnaires that were not included in the final analysis (i.e. those from NHs that were unaware of the option to bill for ACP and those that reported that approval was already planned or in progress) is provided as supplementary material (see Supplementary File 2). 

### Baseline data on respondents and nursing homes

The mean age of respondents was 50.8 years (*SD* = 9.6) in the NH group that had approval for ACP billing and 50.2 years (*SD* = 10.3) in the NH group that did not have such approval. The majority of respondents were female (see Table 1). The largest respondent groups were NH managers and nursing staff managers, with average tenures of 10.8 years (*SD* = 7.7) and 11.8 years (*SD* = 9.3), respectively.

**Table 1:** Respondent characteristics

	Approved nursing homes (*N = * 100)		Non-approved nursing homes (*N = * 68)		*p*-value
**Respondent age (years)**	(*n = * 93)^*^		(*n = * 66)^*^		
Mean (*SD*)	50.8	(9.6)	50.2	(10.3)	0.737
Median (IQR)	54.0	(44.0–59.0)	52.5	(42.75–59.25)	
≤ 40	15	16.1%	14	21.2%	
41–60	69	74.2%	41	62.1%	
> 60	9	9.7%	11	16.7%	
**Respondent sex**	(*n = * 97)^*^		(*n = * 68)^*^		
Male	22	22.7%	24	35.3%	0.191
Female	74	76.3%	43	63.2%	
Diverse	1	1.0%	1	1.5%	
**Respondent position in the nursing home** (multiple answers possible)	(*n = * 99)^*^		(*n = * 68)		
Nursing home manager	56	56.6%	45	66.2%	0.212
Nursing staff manager	27	27.3%	18	26.5%	0.909
Managing director	4	4.0%	12	17.6%	**0.003**
Other (e.g., facilitator)	22	22.2%	5	7.4%	**0.010**
**Years in current position**	(*n = * 97)^*^		(*n = * 67)^*^		
Mean (*SD*)	10.8	(7.7)	11.8	(9.3)	0.696
Median (IQR)	10.0	(4.0–15.0)	8.5	(5.0–18.0)	

^*^Numbers differ due to missing values; *SD* = standard deviation; IQR = interquartile range

No statistically significant differences were observed between NHs that had obtained approval for billing for ACP and those that had not, with respect to respondents’ age, gender, or years in their current position. Of the 27 respondents who selected “other” as their position, 22 were from NHs with approval and 5 were from NHs without approval (22.2% vs. 7.4%). In NHs authorized to bill for ACP, 8 of the 22 questionnaires in this category were completed by ACP facilitators. Other respondents in this group included heads of social services, deputy NH managers, and deputy nursing staff managers.

### Comparison of the characteristics of approved versus non-approved NHs

The majority of NHs operated as non-profit facilities (73.2% of NHs with approval vs. 55.2% of NHs without approval) located in small towns and urban areas (see Table 2). The mean distance to the nearest hospital with an emergency department was 8.0 km (*SD* = 7.0) for NHs with approval and 8.9 km (*SD* = 7.0) for NHs without approval. The mean number of beds was 94.9 (*SD* = 43.1) in NHs with approval and 88.2 (*SD* = 49.1) in NHs without approval. Fewer than half of the responding facilities had a dedicated ward for residents with dementia.

A difference between groups was defined by sponsorship type. NHs that had obtained approval for ACP billing were more likely to be non-profit compared to NHs without such approval (73.2% vs. 55.2%), while private sponsorship was more common among NHs without approval (37.3% vs. 20.6%; *p* = 0.048). No statistically significant differences were observed regarding location, distance to the nearest hospital, facility size (number of beds), or the presence of a dementia-specific ward.

At the nursing staff management level, no significant differences were found in qualifications in end-of-life care, with 68.1% of NHs that had obtained approval and 65.6% of NHs without such approval reporting relevant qualifications. Significant differences emerged at the nursing staff level. The presence of any qualification in end-of-life care was reported more frequently in NHs with approval than in NHs without approval (89.6% vs. 70.1%; *p* = 0.002). Additionally, more nursing staff in NHs with approval for ACP billing had completed advanced palliative care training (160-hour course) (76.0% vs. 49.3%, *p* < 0.001).

A highly significant difference was observed in the availability of ACP, as NHs that had obtained approval for ACP billing were far more likely to provide ACP compared to NHs without such approval (96.0% vs. 36.4%; *p* < 0.001).

**Table 2:** Nursing home characteristics

	Approved nursing homes (*N* = 100)		Non-approved nursing homes (*N* = 68)		*p*-value
**Type of nursing home sponsorship**	(*n* = 97)^*^		(*n* = 67)^*^		
Private	20	20.6%	25	37.3%	**0.048**
Non-profit	71	73.2%	37	55.2%	
Municipal	6	6.2%	5	7.5%	
**Nursing home location**	(*n = * 97)^*^		(*n = * 66)^*^		
Rural (≤ 5,000)	16	16.5%	14	21.2%	0.456
Small town (5,000–20,000)	34	35.1%	25	37.9%	
Semi-urban (20,000–100,000)	21	21.6%	9	13.6%	
Urban (> 100,000)	26	26.8%	18	27.3%	
**Number of beds**	(*n = * 96)^*^		(*n = * 68)^*^		
Mean (*SD*)	94.9	(43.1)	88.2	(49.6)	0.359
Median (IQR)	89.0	(60.75–112.75)	80.0	(60.0–113.25)	
**Presence of a psychogeriatric living area**	(*n* = 99)^*^		(*n = * 68)^*^		
Yes	49	49.5%	27	39.7%	0.212
**Distance to nearest hospital with an emergency department (kilometers)**	(*n* = 71)^*^		(*n* = 57)^*^		
Mean (*SD*)	8.0	(7.0)	8.9	(7.0)	0.405
Median (IQR)	5.0	(2.0–15.0)	8.0	(3.0–15.0)	
**Any qualification in end-of-life care**	(*n* = 91)^*^		(*n* = 64)^*^		
Yes (nursing staff manager)	62	68.1%	42	65.6%	0.744
	(*n* = 96)^*^		(*n* = 67)^*^		
Yes (nursing home staff)	86	89.6%	47	70.1%	**0.002**
**Further training in palliative care (160-hour course)**	(*n* = 96)^*^		(*n* = 67)^*^		
Yes (nursing staff)	73	76.0%	33	49.3%	**< 0.001**
**Any ACP consultations in the nursing home**	(*n* = 100)^*^		(*n* = 66)^*^		
Yes	96	96.0%	24	36.4%	**< 0.001**

^*^Numbers differ due to missing values; *SD* = standard deviation; *IQR* = interquartile range; *ACP* = Advance care planning

### Characteristics of the nursing home residents

The analysis of resident characteristics showed that more than half of the residents in both NH groups had dementia (see Table 3). In addition, 10.5% of residents in NHs with approval for billing ACP and 14.7% of residents in NHs without approval had a diagnosed oncological disease. A high level of care dependency (care grade 4 or 5) was observed in 41.4% of residents in NHs with approval and 37.4% in NHs without approval, with smaller proportions being completely bedridden. Furthermore, 35.9% of residents in NHs with approval and 41.2% in NHs without approval had experienced at least one hospitalization during the past year. Overall, there were no statistically significant differences in resident characteristics between NHs that had obtained approval for ACP billing and those that had not.

**Table 3:** Nursing home resident characteristics

Proportion of residents…	Approved nursing homes (*N = * 100)	Non-approved nursing homes (*N = * 68)	*p*-value
…with a dementia diagnosis Mean (*SD*)	(*n* = 94)^*^ 59.0% (19.0)	(*n* = 67)^*^ 54.3% (22.6)	0.171
…with an oncological diagnosis Mean (*SD*)	(*n* = 88)^*^ 10.5% (9.8)	(*n* = 64)^*^ 14.7% (14.9)	0.086
…with a care grade 4 or 5^**^ Mean (*SD*)	(*n* = 94)^*^ 41.4% (16.6)	(*n* = 64)^*^ 37.4% (23.8)	0.112
…being entirely bedridden Mean (*SD*)	(*n* = 86)^*^ 9.4% (14.8)	(*n* = 63)^*^ 6.8% (7.7)	0.969
…with at least one hospital admission in 2022 Mean (*SD*)	(*n* = 83)^*^ 35.9% (21.9)	(*n* = 59)^*^ 41.2% (25.6)	0.228

^*^Numbers differ due to missing values; ^**^according to the German compulsory nursing care insurance scheme; care grade 1: minor limitations in independence or skills; care grade 2: significant limitations in independence or skills, care grade 3: severe limitations in independence or skills; care grade 4: extremely severe limitations in independence or skills; care grade 5: extremely severe limitations in independence or skills, with special care requirements; *SD* = standard deviation

### ACP, advance directives, and care preferences at the end of life

NHs reported that at least one ACP consultation had been conducted for 42.0% of residents in NHs that had obtained approval for ACP billing and for 36.3% in NHs without such approval (see Table 4). The proportion of residents with a written health care proxy was significantly higher in NHs with approval (66.7% vs. 58.7%, *p* = 0.049), as was the proportion of residents with a written advance directive (68.5% vs. 55.6%, *p* < 0.001). In contrast, plans for emergency situations (i.e., acute, major health crises involving incapability to consent to medical procedures) were less common, reported for 36.0% of residents in NHs with approval and 37.6% in NHs without approval, with no significant difference between the two groups.

The perceived meaningfulness of available documents—specifically regarding hospital transfer in the last phase of life or care preferences in the event of cardiac arrest—did not differ significantly between NHs that had obtained approval for billing ACP and those that had no such approval. Documents were considered meaningful for 52.8% of residents in NHs with approval and 44.3% in NHs without approval in the context of a hospital transfer. For cardiac arrest, the perceived meaningfulness was slightly higher, at 59.5% and 45.6%, respectively.

**Table 4:** Advance care planning, advance directives, and care preferences at the end of life

Proportion of residents…	Approved nursing homes (*N = * 100)	Non-approved nursing homes (*N = * 68)	*p*-value
…with at least one ACP consultation Mean (*SD*)	(*n* = 91)^*^ 42.0% (33.5)	(*n* = 23)^*^ 36.3% (31.8)	0.446
…with a health care proxy (written) Mean (*SD*)	(*n* = 87)^*^ 66.7% (28.9)	(*n* = 60)^*^ 58.7% (27.9)	**0.049**
…with an advance directive (written) Mean (*SD*)	(*n* = 93)^*^ 68.5% (21.5)	(*n* = 62)^*^ 55.6% (21.9)	**< 0.001**
…with a POLST (written) Mean (*SD*)	(*n* = 69)^*^ 36.0% (37.7)	(*n* = 50)^*^ 37.6% (40.7)	0.782
**Proportion of residents for whom the above documents were considered meaningful in terms of…**			
…hospital transfer in the last phase of life Mean (*SD*)	(*n* = 84)^*^ 52.8% (37.5)	(*n* = 60)^*^ 44.3% (38.5)	0.133
…care preferences in the event of cardiac arrest Mean (*SD*)	(*n* = 85)^*^ 59.5% (37.0)	(*n* = 56)^*^ 45.6% (38.2)	0.056

^*^ Numbers differ due to missing values; *ACP* = advance care planning; *SD* = standard deviation;

*POLST *= plan for emergency situations (documented on a “Physician’s Order for Life-Sustaining Treatment”)

### Cooperation in end-of-life care

**Fig. 2:**
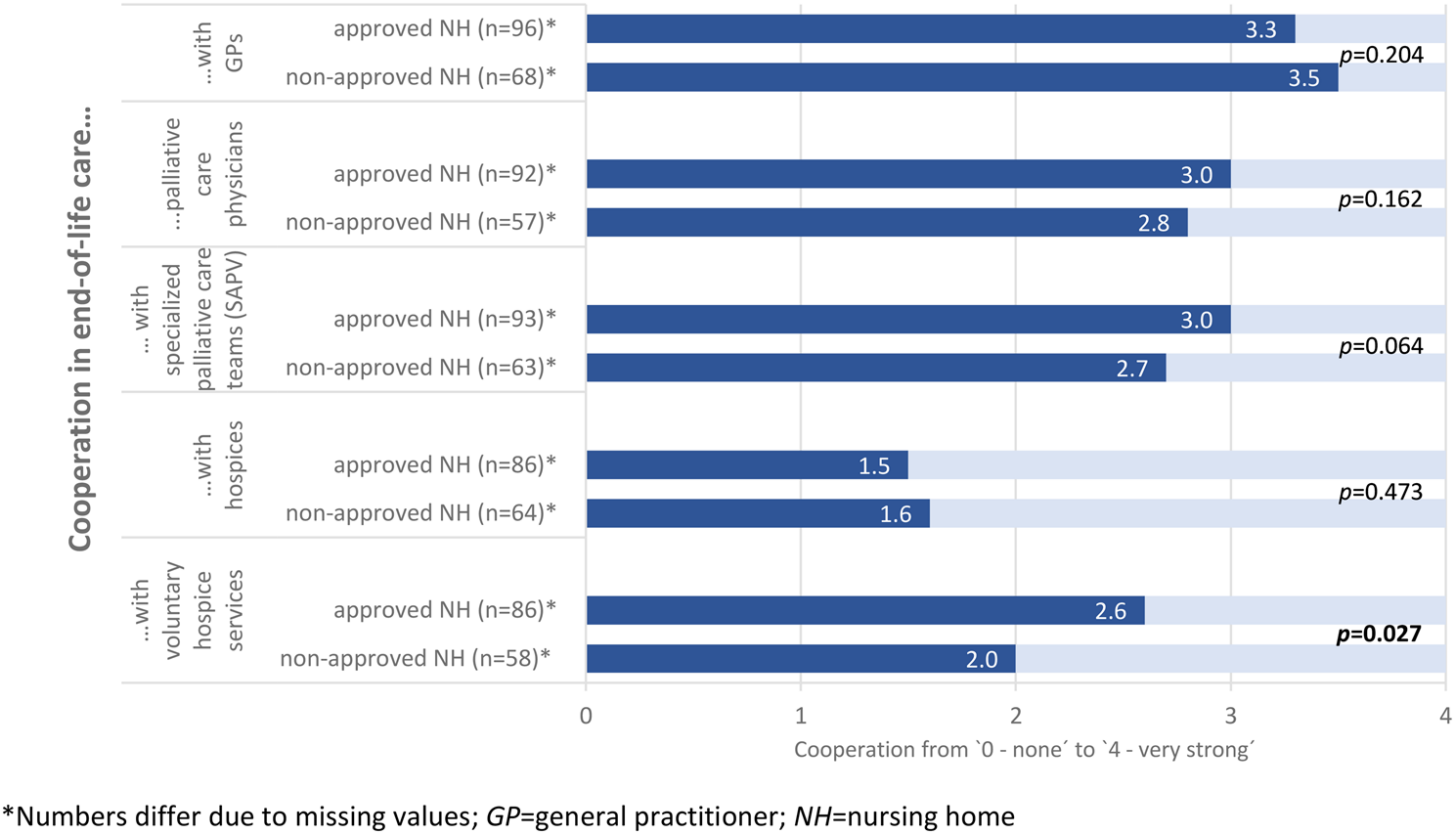
Cooperation in end-of-life care.

The highest levels of collaboration with external health care providers in end-of-life care were reported for general practitioners (GPs), with mean scores of 3.3 in NHs that had obtained approval for billing ACP and 3.5 in NHs without approval, on a scale ranging from 0 to 4 (see Fig. 2). In contrast, cooperation with inpatient hospices received the lowest ratings, with mean scores of 1.5 and 1.6, respectively. There were no significant differences between NHs with and without approval regarding collaboration with GPs, palliative care physicians, specialized palliative care teams, or hospices. Cooperation with voluntary hospice services was rated significantly higher in NHs that had obtained approval for ACP billing (2.6 vs. 2.0, *p* = 0.027).

## Discussion

The present cross-sectional study compared NHs that had obtained approval under § 132g SGB V to bill for ACP consultations with those that had not obtained approval and had no intention of doing so. No significant differences were found in resident characteristics between the two groups. Overall, few significant differences emerged in the comparison of NHs. While NHs with approval for billing ACP had a higher proportion of residents with written health care proxies and advance directives, they did not show a significantly greater proportion of residents with emergency plans. Additionally, although a slightly higher proportion of available documents was deemed useful in the event of end-of-life hospital transfer or cardiac arrest in NHs with approval for ACP billing, this difference did not reach statistical significance. At the facility level, differences were observed in sponsorship type, staff qualifications in end-of-life care, and collaboration with voluntary hospice services.

### Qualification and collaboration in end-of-life care

The higher qualifications in end-of-life care observed among staff in NHs that had obtained approval for ACP billing align with key strategies for enhancing end-of-life care. As NHs are increasingly serving as prominent settings for end-of-life care and death [5–7], national and international initiatives are aiming to foster greater hospice and palliative care cultures within these facilities [24, 25]. Such initiatives emphasize training, capacity building, staff support, and ACP, all of which are considered essential components of palliative care programs in NHs [26]. In Germany, approval for ACP billing under statutory health insurance requires NHs to implement a palliative care concept that incorporates ACP into the overarching organizational structure [17]. Given this requirement, it is unsurprising that NHs with approval for billing for ACP demonstrate greater expertise in hospice and palliative care. This may indicate a stronger palliative care culture, as postulated by Walther et al. (2022) based on their findings [27].

A similar pattern was observed in the stronger collaboration between NHs that had obtained approval for ACP billing and voluntary hospice services. In these NHs, cooperation with voluntary hospice providers was comparable to that with professional palliative care teams and physicians. Compared to NHs that had not received approval for ACP billing and had no intention of applying for it, NHs with approval for billing appeared to have more extensive networks of external palliative care providers. This aligns with networking as a prerequisite for ACP billing [17], and may further indicate an established hospice and palliative care culture, characterized by a heightened awareness that effective palliative care requires a multidisciplinary approach [24, 28].

However, no differences were found between the NH groups in terms of their cooperation with GPs, palliative care physicians, or specialized palliative care teams. In the context of end-of-life care, the strongest collaboration was reported with GPs. Cooperation with palliative care physicians or specialized palliative care teams was rated as less strong in both groups. This is in line with the nature of outpatient primary care and primary palliative care provided by GPs in NHs and the regular contact between almost all residents and their GPs, as described in several studies [29–31]. The intended networking with external palliative care providers as part of the requirements for ACP billing does not therefore appear to have an impact on closer cooperation between NHs and inpatient hospices in end-of-life care.

### Advance care planning and the availability of precautionary documents

As anticipated, the present study identified a significant discrepancy in the prevalence of ACP consultations between NHs that had obtained approval for ACP billing and those that had not. However, the results also demonstrated that not all NHs with approval for ACP billing provided ACP consultations, while more than one-third of NHs without such approval did offer ACP. In the latter cases, it was unclear who delivered ACP and to what extent these individuals were qualified to perform such tasks. Conversely, some NHs appeared unable to offer ACP to their residents, despite being authorized to bill for it. The reasons behind this inability to offer ACP were not captured in the data, but this finding may point to limitations in the current strategy of ACP implementation. In many NHs, ACP is facilitated through a single staff member who has undergone the required training to serve as an ACP facilitator [22, 32]. Consequently, if this facilitator leaves the NH or is absent for an extended period due to illness, ACP services may no longer be available, despite the NH’s ability to bill for them [33].

A comparison at the resident level between NHs with and without billing approval revealed that the proportion of residents who had at least one ACP consultation was slightly higher in NHs with approval for ACP billing, although the difference was not statistically significant. Moreover, in both groups, less than half of the residents had participated in an ACP consultation. This finding highlights two key aspects: (1) a considerable number of NHs offered ACP consultations outside of formal reimbursement structures; and (2) even in NHs that had obtained approval for ACP billing, only a subset of residents received ACP consultations. This result aligns with previous studies indicating that many NH residents do not receive or engage in ACP due to factors such as dementia, generally poor health, staff shortages, a lack of qualified staff, and personal preferences [34–36].

The primary objective of ACP is to empower residents to articulate their preferences for future medical treatment and care, ultimately facilitating the creation of effective advance directives [15]. Consequently, the production of precautionary documents—particularly physician treatment orders for emergency situations and advance directives—serves as a key indicator of successful ACP [37] and is frequently recorded as an outcome measure in studies evaluating ACP implementation [38, 39]. In the present study, NHs that had obtained approval for ACP billing under § 132g had a significantly higher prevalence of written health care proxies and advance directives compared to NHs without approval for ACP billing. This aligns with the results of international intervention studies, which have reported an increase in precautionary document completion following the implementation of ACP programs in NHs [40–42]. However, no significant difference was observed in the proportion of residents with a documented plan for emergency situations between the two groups. Given that structured ACP implementation is likely to support the systematic preparation of advance care documents, it was anticipated that NHs that had obtained approval for ACP billing would demonstrate a higher prevalence of emergency plans. Thus, the lack of statistical difference was surprising. However, potential explanations for the finding may be offered by a systematic review suggesting that not all formal ACP consultations result in advance care documents, even in cases where residents discuss their treatment and care preferences; and that emergency plans may be disproportionately created for only the most critically ill residents [39].

Moreover, the present analysis revealed no statistically significant difference in the informative value of precautionary documents between NHs with and without approval for ACP billing in the context of hospital transfer at the end of life or cardiac arrest, though a higher trend was observed in NHs that had obtained approval. Given the qualification requirements for ACP facilitators under § 132 g, it was expected that documents created within these regulated ACP consultations would be rated as more informative than those from NHs offering ACP outside these regulations. This result may also reflect the overall low prevalence of emergency plans in both NH groups. However, such plans are essential for ensuring that clinical decision-making serves the interests of residents in both hospital transfer and cardiac arrest scenarios.

The proportion of residents in both NH groups with health care proxies and advance directives exceeded the proportion of those who had received at least one ACP consultation. This suggests that some residents entered the NH with pre-existing precautionary documents. Findings from a representative population survey conducted in Germany support this conclusion, as more than one-third of respondents had already drafted an advance directive, while another third planned to do so in the near future [43]. Similarly, a systematic review revealed that the majority of NH residents already had pre-existing care plans at the time of admission [44]. The review authors suggested that residents with pre-existing documents may have perceived ACP as redundant, leading to reduced engagement in ACP consultations [44]. However, the results also suggested that advance directives drafted prior to NH admission may not fulfil their intended purpose [43]. On average, these documents were 6 years old, and in most cases, they were prepared using solely internet resources. Only approximately a quarter of respondents had sought professional assistance from lawyers or physicians, suggesting that many directives had been prepared without expert legal or medical input. Nevertheless, most respondents believed their advance directives would be honored in practice [43]—a belief not supported by the present results regarding the informative value of the documents available at NHs.

For advance directives to effectively uphold self-determination in cases of incapacity, they must be clinically meaningful and applicable to relevant medical scenarios [15]. The present results highlight potential for improvement, even among NHs offering ACP under § 132 g SGB V.

### Strengths and limitations

The study sample included NHs from all German federal states and of varying sizes. The mean NH size (based on the number of beds), sponsorship distribution, and average distance to the nearest hospital closely align with figures reported in other German NH studies [24, 27, 45, 46]. Although the overall response rate was slightly lower than in comparable NH surveys [45, 46], the NHs included in this study are likely representative of NHs across Germany.

Due to the specific research focus of the subgroup analysis, approximately half of the initially collected questionnaires were excluded from the final analysis. While this approach enabled a clear differentiation of NHs for comparison, it also limited the sample size available for analysis. Despite the fact that only 15.3% (1,773) of all NHs in Germany obtained approval for ACP billing [18], 30.3% of those responding to the study had such authorization. This allowed for valuable insights into the characteristics that distinguish NHs that had obtained approval for ACP billing from the majority of NHs in Germany without such approval.

A potential response bias cannot be ruled out, as most respondents were NH managers or nursing staff managers, and these figures may have attempted to present their NHs in a favorable light. There may also be a bias in the responses regarding the usefulness of the precautionary documents in the NHs in cases of hospitalization or cardiac arrests, as these are medical issues and the questionnaire did not provide any information on which basis the assessments should be made. Furthermore, we cannot verify whether the respondents, who were mostly management staff, had consulted medical staff when answering the questions. However, given the anonymous nature of the survey, it is reasonable to assume that respondents answered to the best of their knowledge and were not systematically motivated to provide overly positive portrayals. Additionally, respondents in management positions may have a broader overview of potential problems and shortcomings when dealing with precautionary documents that exists for the residents of their facility. The estimates collected in the questionnaire regarding resident characteristics, including information on hospital admissions, are also subject to the aforementioned limitations and should therefore be interpreted with caution, as it is unknown to what extent the figures given in the answers were substantiated using data from the NH’s documentation. However, the estimated proportions of residents with a dementia diagnosis or who are entirely bedridden are close to the results of a nationwide survey by Fassmer et al. [46]. This survey was also based on information from NH management on the characteristics of NH residents. Furthermore, the results regarding the proportion of residents with care level 4 or 5 are close to the figures obtained in a 2022 study conducted in German nursing homes by trained study nurses on the basis of nursing records [30]. Therefore, the estimated figures are considered plausible.

With regard to questions concerning cooperation with external providers in end-of-life care, there may be overlaps between GPs, palliative care physicians, and specialized palliative care teams, as GPs may also work as palliative care physicians and may then belong to a specialized palliative care team, although these role overlaps are not necessarily the case. Consequently, in consultation with the project advisory board, we decided to reflect this diversity by asking separately about collaboration with GPs, palliative care physicians, and specialist palliative care teams. The comparatively higher number of missing responses regarding cooperation with palliative care physicians may be due to this overlap in roles. Similarly, the higher number of missing values in some questions about cooperation between NHs and hospices or voluntary hospice services could indicate that cooperation is not yet well established in individual NHs.

## Conclusions

ACP appears to be offered in both NHs that bill for ACP consultations and those that do not, although overall resident participation remains low. While differences in palliative care structures, such as staff qualifications and structured ACP services, are evident, their impact on ACP consultation rates and the creation of precautionary documents is less pronounced than expected. The limited availability of emergency plans, combined with the lack of meaningfulness of a substantial proportion of advance directives and associated documents—even those created under § 132g SGB V—raises concerns about the quality of advance care directives as an outcome of ACP. The extent to which NH residents’ advance directives are created as part of comprehensive ACP remains unclear, as does the extent to which residents possess advance directives prior to NH admission and the extent to which these documents are adapted to the NH setting and residents’ current health conditions.

To strengthen ACP implementation in NHs, further research is needed to gain deeper insight into ACP services, develop quality assurance measures for ACP consultations and documents, and evaluate (in a results-oriented manner) whether the documented preferences of NH residents are ultimately honored. There is also an urgent need to evaluate the extent to which advance directives can contribute to reducing unnecessary, burdensome treatments in the final months of life. 

## Supplementary information

Below is the link to the electronic supplementary material.

Supplementary Material 1

## Data Availability

The datasets used and/or analyzed during the present study are available from the corresponding author upon reasonable request.

## Abbreviations

NH: Nursing home
ACP: Advance care planning
SGB V: German Social Code, Book V
SD: Standard deviation
IQR: Interquartile range
GP: General practitioner
