# Supplementary material for: To bill or not to bill – a cross-sectional study comparing funded and unfunded advance care planning services in German nursing homes
Source: BMC Health Serv Res. 2025 Dec 25;26:139. doi: 10.1186/s12913-025-13848-6 (PMC12849682; doi:10.1186/s12913-025-13848-6)
Supplement: Supplementary file 1 — Supplementary Material 1 [file 12913_2025_13848_MOESM1_ESM.docx]

**Supplementary Material 1** Nursing home characteristics

|  | Approved NHs (*N*=100) | | Non-approved NHs (*N*=68) | | NHs unaware of billing options (*N*=111) | | NHs with approval in planning or who have already applied for (*N*=27) | |
| --- | --- | --- | --- | --- | --- | --- | --- | --- |
| **Type of nursing home sponsorship** | (*n*=97)^*^ | | (*n*=67)^*^ | | (*n*=108)^*^ | | (*n*=26)^*^ | |
| Private | 20 | 20.6% | 25 | 37.3% | 56 | 51.9% | 10 | 38.5% |
| Non-profit | 71 | 73.2% | 37 | 55.2% | 45 | 41.7% | 15 | 57.7% |
| Municipal | 6 | 6.2% | 5 | 7.5% | 7 | 6.5% | 1 | 3.8% |
| **Nursing home location** | (*n=*97)^*^ | | (*n=*66)^*^ | | (*n*=106)^*^ | | (*n*=25)^*^ | |
| Rural (≤5,000) | 16 | 16.5% | 14 | 21.2% | 23 | 21.7% | 2 | 8.0% |
| Small town (5,000–20,000) | 34 | 35.1% | 25 | 37.9% | 44 | 41.5% | 10 | 40.0% |
| Semi-urban (20,000–100,000) | 21 | 21.6% | 9 | 13.6% | 24 | 22.6% | 5 | 20.0% |
| Urban (>100,000) | 26 | 26.8% | 18 | 27.3% | 15 | 14.2% | 8 | 32.0% |
| **Number of beds** | (*n=*96)^*^ | | (*n=*68)^*^ | | (*n*=108)^*^ | | (*n*=26)^*^ | |
| Mean (*SD*) | 94.9 | (43.1) | 88.2 | (49.6) | 75.3 | (35.2) | 93.9 | (43.2) |
| Median (IQR) | 89.0 | (60.75–112.75) | 80.0 | (60.0–113.25) | 69.5 | (49.5–94.75) | 85.0 | (60.75–106.0) |
| **Presence of a psychogeriatric living area** | (*n*=99)^*^ | | (*n=*68)^*^ | | (*n*=110)^*^ | | (*n*=26)^*^ | |
| Yes | 49 | 49.5% | 27 | 39.7% | 41 | 37.3% | 11 | 42.3% |
| **Distance to nearest hospital with an emergency department (kilometers)** | (*n*=71)^*^ | | (*n*=57)^*^ | | (*n*=95)^*^ | | (*n*=24)^*^ | |
| Mean (*SD*) | 8.0 | (7.0) | 8.9 | (7.0) | 9.7 | (7.3) | 6.9 | (8.2) |
| Median (IQR) | 5.0 | (2.0–15.0) | 8.0 | (3.0–15.0) | 10.0 | (2.5–15.0) | 3.0 | (1.5–11.0) |
| **Any qualification in end-of-life care** | (*n*=91)^*^ | | (*n*=64)^*^ | | (*n*=107)^*^ | | (*n*=25)^*^ | |
| Yes (nursing staff manager) | 62 | 68.1% | 42 | 65.6% | 40 | 37.4% | 15 | 60.0% |
|  | (*n*=96)^*^ | | (*n*=67)^*^ | | (*n*=109)^*^ | | (*n*=23)^*^ | |
| Yes (nursing home staff) | 86 | 89.6% | 47 | 70.1% | 65 | 59.6% | 20 | 87.0% |
| **Further training in palliative care (160-hour course)** | (*n*=96)^*^ | | (*n*=67)^*^ | | (*n*=109)^*^ | | (*n*=23)^*^ | |
| Yes (nursing staff) | 73 | 76.0% | 33 | 49.3% | 42 | 38.5% | 16 | 69.6% |
| **Any ACP consultations in the nursing home** | (*n*=100)^*^ | | (*n*=66)^*^ | | (*n*=106)^*^ | | (*n*=26)^*^ | |
| Yes | 96 | 96.0% | 24 | 36.4% | 25 | 23.6% | 10 | 38.5% |

^*^Numbers differ due to missing values; *NHs*=Nursing homes; *SD*=standard deviation; *IQR*=interquartile range; *ACP*=Advance care planning

**Supplementary Material 2** Nursing home resident characteristics

| Proportion of residents… | Approved NHs (*N=*100) | Non-approved NHs (*N=*68) | NHs unaware of billing options (*N*=111) | NHs with approval in planning or who have already applied for (*N*=27) |
| --- | --- | --- | --- | --- |
| …with a dementia diagnosis  Mean (*SD*) | (*n*=94)^*^  59.0% (19.0) | (*n*=67)^*^  54.3% (22.6) | (*n*=110)^*^  54.6% (24.1) | (*n*=27)^*^  52.1% (22.2) |
| …with an oncological diagnosis  Mean (*SD*) | (*n*=88)^*^  10.5% (9.8) | (*n*=64)^*^  14.7% (14.9) | (*n*=103)^*^  10.2% (10.6) | (*n*=26)^*^  7.1% (6.3) |
| …with a care grade 4 or 5^**^  Mean (*SD*) | (*n*=94)^*^  41.4% (16.6) | (*n*=64)^*^  37.4% (23.8) | (*n*=110)^*^  36.4% (19.9) | (*n*=26)^*^  34.3% (17.7) |
| …being entirely bedridden  Mean (*SD*) | (*n*=86)^*^  9.4% (14.8) | (*n*=63)^*^  6.8% (7.7) | (*n*=105)^*^  7.0% (9.3) | (*n*=26)^*^  4.8% (4.6) |
| …with at least one hospital admission in 2022  Mean (*SD*) | (*n*=83)^*^  35.9% (21.9) | (*n*=59)^*^  41.2% (25.6) | (*n*=102)^*^  40.8% (15.3) | (*n*=26)^*^  34.3% (22.5) |

^*^Numbers differ due to missing values; ^**^according to the German compulsory nursing care insurance scheme; care grade 1: minor limitations in independence or skills; care grade 2: significant limitations in independence or skills, care grade 3: severe limitations in independence or skills; care grade 4: extremely severe limitations in independence or skills; care grade 5: extremely severe limitations in independence or skills, with special care requirements; *NHs*=Nursing homes; *SD*=standard deviation

**Supplementary Material 3** Advance care planning, advance directives, and care preferences at the end of life

| Proportion of residents… | Approved NHs (*N=*100) | Non-approved NHs (*N=*68) | NHs unaware of billing options (*N*=111) | NHs with approval in planning or who have already applied for (*N*=27) | |
| --- | --- | --- | --- | --- | --- |
| …with at least one ACP consultation  Mean (*SD*) | (*n*=91)^*^  42.0% (33.5) | (*n*=23)^*^  36.3% (31.8) | (*n*=25)^*^  39.3% (36.9) | (*n*=10)^*^  34.7% (33.8) | |
| …with a health care proxy (written)  Mean (*SD*) | (*n*=87)^*^  66.7% (28.9) | (*n*=60)^*^  58.7% (27.9) | (*n*=98)^*^  66.4% (29.2) | (*n*=25)^*^  62.4% (29.9) | |
| …with an advance directive (written)  Mean (*SD*) | (*n*=93)^*^  68.5% (21.5) | (*n*=62)^*^  55.6% (21.9) | (*n*=107)^*^  56.3% (28.6) | (*n*=25)^*^  59.3% (25.0) | |
| …with a POLST (written)  Mean (*SD*) | (*n*=69)^*^  36.0% (37.7) | (*n*=50)^*^  37.6% (40.7) | (*n*=74)^*^  27.5% (38.0) | (*n*=18)^*^  36.4% (38.6) | |
| Proportion of residents for whom the above documents were considered meaningful in terms of… | | | | |  |
| …hospital transfer in the last phase of life  Mean (*SD*) | (*n*=84)^*^  52.8% (37.5) | (*n*=60)^*^  44.3% (38.5) | (*n*=97)^*^  49.0% (37.1) | (*n*=25)^*^  53.2% (40.4) | |
| …care preferences in the event of cardiac arrest  Mean (*SD*) | (*n*=85)^*^  59.5% (37.0) | (*n*=56)^*^  45.6% (38.2) | (*n*=95)^*^  52.3% (36.3) | (*n*=23)^*^  60.0% (34.8) | |

^*^ Numbers differ due to missing values; *NHs*= Nursing homes; *ACP*=advance care planning; *SD*=standard deviation;

POLST=plan for emergency situations (documented on a “Physician’s Order for Life-Sustaining Treatment”)

**Supplementary Material 4** Cooperation in end-of-life care

*Numbers differ due to missing values; *GP*=general practitioner; *NHs*=nursing homes
